# A tribute to Ko Shimamoto (1949–2013)

**DOI:** 10.1093/jxb/eru104

**Published:** 2014-03-18

**Authors:** Paula Suárez-López, Hiroyuki Tsuji, George Coupland

**Affiliations:** ^1^Centre for Research in Agricultural Genomics, CSIC-IRTA-UAB-UB, Campus UAB, Bellaterra (Cerdanyola del Vallès), 08193 Barcelona, Spain; ^2^Laboratory of Plant Molecular Genetics, Graduate School of Biological Sciences, Nara Institute of Science and Technology, 8916-5 Takayama, Ikoma, Nara 630-0192, Japan; ^3^Max Planck Institute for Plant Breeding Research, Carl von Linné Weg 10, D-50829 Cologne, Germany

**Figure:**
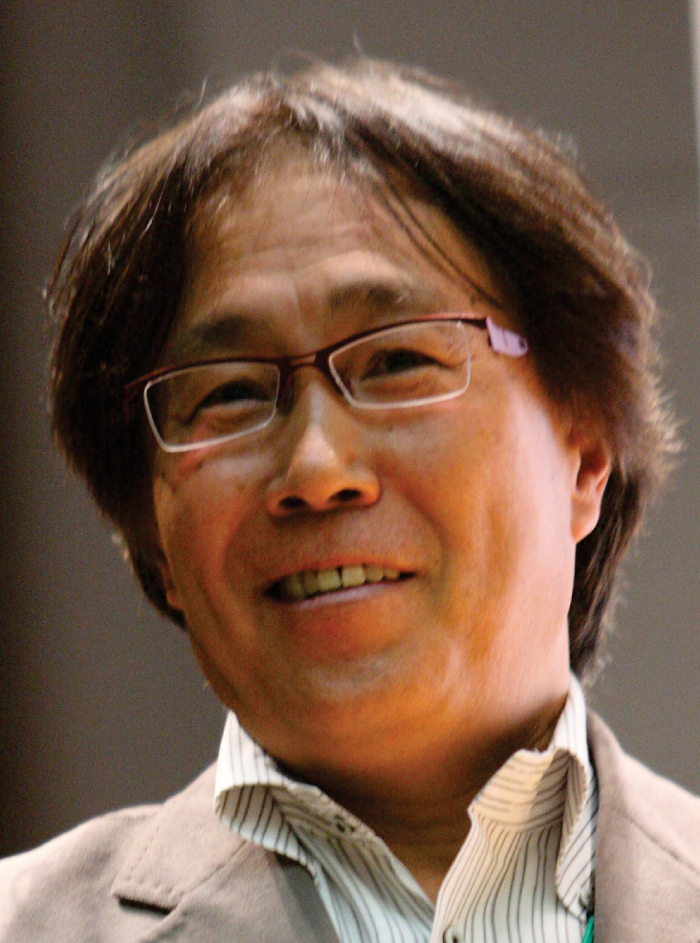
Professor Ko Shimamoto (1949–2013)

Professor Ko Shimamoto tragically passed away on 28 September 2013, at the age of 63, after an illness. He was born in October 1949 in Wakayama Prefecture, a warm southern area near Nara where he ultimately established his laboratory. He grew up in Toyohashi city near Nagoya, in the central part of Japan. ‘Because Ko and I shared the same hometown’ — evokes Hiroyuki Tsuji — ‘we sometimes enjoyed talking about changes in the architecture in these cities’.

Ko graduated from the Department of Agriculture at Kyoto University and then moved to the USA to become a graduate student at the University of Wisconsin-Madison in 1974. After receiving his PhD degree in genetics in 1980, he started postdoctoral training at the Friedrich Miescher Institute, Basel, Switzerland. In 1984, he returned to Japan to join the Plantech Research Institute, which was established by the Mitsubishi Chemical Corporation, initiating a programme using biotechnology in rice breeding. He immediately began experimenting with conditions for rice transformation, and in 1989 he succeeded in regenerating the first fertile transgenic rice plants from protoplasts (Shimamoto *et al.*, 1989). This achievement resulted from his highly sophisticated tissue culture techniques that were continuously perfected during his research career. He generated several new cultivars of rice by means of protoplast culture and regeneration, putting his research group at the forefront of rice breeding. In 1993, he moved to the newly established Nara Institute of Science and Technology and started his own new laboratory in the Graduate School of Biological Sciences as a Professor of Plant Molecular Genetics. He launched three different themes in the new laboratory, plant innate immunity, gene silencing, and flowering, and each was full of exciting achievements. With these choices, Ko devoted his scientific life to understand traits with enormous relevance for agriculture and to develop tools that could be useful for rice improvement.

## Plant innate immunity

Ko’s laboratory shed light on the mechanisms of plant immunity in rice. When pathogens infect plants, pathogen-derived molecules are recognized by pattern recognition receptors at the cell surface, triggering a signalling cascade that leads to protection against the pathogen (reviewed by Kawano and Shimamoto, 2013). An early response is the production of reactive oxygen species (ROS), which can be toxic for pathogens and also induce plant cell death at the site of infection, an effect known as the hypersensitive response (reviewed by Senthil-Kumar and Mysore, 2013). At the turn of the century, Ko’s group described that the small GTPase Rac1 enhances resistance to two pathogens, rice blast fungus and bacterial blight. Through activation of NADPH oxidase, Rac1 increases the production of ROS and regulates cell death in rice (Kawasaki *et al.*, 1999; Ono *et al.*, 2001). Since then, his laboratory has made remarkable contributions to the dissection of this defence pathway, identifying numerous Rac1-interacting proteins as well as upstream regulators and downstream targets.

Ko’s group characterized at least three upstream regulators of Rac1 in the response to fungal pathogens: a subunit of the heterotrimeric G protein (Dwarf1), which is an important signal transduction complex in animals and plants (Suharsono *et al.*, 2002); serotonin, a well-known animal neurotransmitter, which plays a role in plant defence in a process mediated by Rac1 and Dwarf1 (Fujiwara *et al.*, 2010); and Pit, a nucleotide-binding domain and leucine-rich repeat-containing protein that affects ROS production and the hypersensitive response (Kawano *et al.*, 2010).

Step by step, he described interactions of Rac1 with other proteins involved in plant immunity. These Rac1-interacting factors include proteins with a range of functions, such as kinases, receptor kinases, two guanine nucleotide exchange factors that activate Rac1, chaperones and co-chaperones, a transcription factor, and a receptor for protein kinase C that in animals acts as a scaffolding protein with important roles in development, ageing, cancer, and various signalling processes (Akamatsu *et al.*, 2013; Chen *et al.*, 2010; Lieberherr *et al.*, 2005; Nakashima *et al.*, 2008; Thao *et al.*, 2007; Wamaitha *et al.*, 2012; Yamaguchi *et al.*, 2012; Yamaguchi *et al.*, 2013). These interactions, detected by diverse methods, were confirmed through proteomic analyses, which identified additional Rac1-interacting proteins (Fujiwara *et al.*, 2009; Fujiwara *et al.*, 2006; Tsunezuka *et al.*, 2005). The work of his group led to the functional characterization of many of these Rac1 interactors, establishing their specific roles in defence signalling. Altogether, these results indicate the formation of protein complexes regulating innate immunity in rice.

During the last few years, the work of Ko’s laboratory showed that a membrane-bound chitin receptor (CERK1) triggers a signal transduction cascade in response to pathogen-derived chitin. This cascade rapidly activates Rac1 and induces defence responses. CERK1 binds several Rac1-interacting proteins in the endoplasmic reticulum and this complex seems to traffic from the endoplasmic reticulum to the plasma membrane, where the complex interacts with Rac1 (Akamatsu *et al.*, 2013; Chen *et al.*, 2010; Yamaguchi *et al.*, 2013). The work of Akamatsu *et al.* (2013) includes an illustrative model of the major findings of Ko’s group in the field of plant immunity.

Downstream effectors of Rac1 were also identified by Ko’s laboratory. Rac1 downregulates a metallothionein, MT2b, which acts as an ROS scavenger (Wong *et al.*, 2004). Together with the role of Rac1 on NADPH regulation, this suggests that Rac1 induces ROS production and suppresses ROS scavenging, making a double contribution to the oxidative burst associated with disease resistance. Later work of Ko’s group showed that the activation of ROS production results from direct interaction between Rac1 and the catalytic subunit of NADPH oxidase (Rboh) at the plasma membrane (Wong *et al.*, 2007). Rac1 also activates an enzyme involved in lignin biosynthesis, leading to lignin accumulation (Kawasaki *et al.*, 2006). This also contributes to plant defence, as lignin acts as a physical barrier against pathogens. A basic helix-loop-helix transcription factor, RAI1 (Rac Immunity1), is another Rac1-downstream factor, which is phosphorylated by kinases present in the Rac1 complex and increases resistance to blast fungus (Kim *et al.*, 2012).

His last published work is a collaboration with the laboratory of Chojiro Kojima reporting the crystallization of Rac1 and a preliminary X-ray crystallographic analysis to determine Rac1 structure (Kosami *et al.*, 2014).

Therefore, Ko made significant advances in the molecular mechanisms underlying the response of rice to pathogens. He also helped to understand the roles that proteins with well-known functions in animals play in plants.

## Flowering

Ko also made major contributions to understanding the molecular control of photoperiodic flowering. His early work related to understanding the short-day flowering response in rice, and then later broadened to study the role of FT-like proteins as systemic signals in diverse photoperiodic responses. His first important contribution was the demonstration that the photoperiodic flowering pathways of *Arabidopsis* and rice involve similar components functioning in the same order, but which confer the opposite response to day length (Hayama *et al.*, 2003). *Arabidopsis* is a long-day plant and is therefore induced to flower under long days, whereas these conditions repress flowering in rice. At this time, CONSTANS (CO) transcription factor had been shown to have a central role in the photoperiodic response of *Arabidopsis* by activating transcription of *FLOWERING LOCUS T* (*FT*) in response to long days, and CO transcription had been shown to be regulated by GIGANTEA (GI). Furthermore, the *CO* homologue *HEADING DATE 1* (*Hd1*) and the *FT* homologue *HEADING DATE 3a* (*Hd3a*) had been identified in rice as quantitative trait loci contributing to photoperiodic flowering. However, how these proteins could confer long-day flowering in *Arabidopsis* but short-day flowering in rice was unclear. Ko’s group showed that overexpression of the rice homologue of *GI* (*OsGI*) in transgenic rice plants caused increased expression of *Hd1*, but that in contrast to *Arabidopsis* this was associated with late flowering and reduced *Hd3a* expression. Transient expression assays demonstrated that Hd1 acted as a transcriptional repressor of *Hd3a*. Thus, they proposed that the photoperiodic response of rice involves transcriptional repression of *Hd3a* by Hd1 under long days, but that this does not occur under short days, so flowering proceeds under these conditions. This influential model suggested that the core photoperiodic flowering pathway involving GI–CO–FT is highly conserved among flowering plants, but that changes in the connectivity between components are responsible for evolution of photoperiodic responses. More recently, many more rice flowering-time genes have been identified adding complexity, but the connections identified by Ko’s group remain central to these models.

The work of Hayama *et al.* (2003) demonstrated that activation of *Hd3a* transcription under short days was the photoperiodic output that led to early flowering. This result stimulated Ko to study more thoroughly the role of FT-like proteins in flowering of rice, and his group showed that Hd3a and the related protein RICE FLOWERING LOCUS T1 (RFT1) are essential for rice flowering, because the double mutant *hd3a rft* does not flower under long or short days (Komiya *et al.*, 2008). This result contrasts with *Arabidopsis*, where double mutants of *FT* and its homologue *TWIN SISTER OF FT* are day-length insensitive, but still able to flower. The idea that Hd3a and RFT are so closely associated with flowering in rice inspired Ko to focus his group on understanding their function in flowering-time control. By this time, it had been shown in *Arabidopsis* that *FT* promotes flowering when transcribed in the leaf vasculature, but that FT protein acts at the shoot apical meristem by interacting with the bZIP transcription factor FLOWERING LOCUS D (FD), and that the floral promotive activity of *FT*-like genes can cross graft junctions in *Arabidopsis* and tomato. As reviewed in detail elsewhere (Kobayashi and Weigel, 2007; Turck *et al.*, 2008), there was speculation based on these observations that a product of FT might represent the graft transmissible substance florigen, the long-sought after signal made in the leaves that induces flower development at the shoot apex. Ko’s group aimed to test directly whether Hd3a protein was able to move from the leaves to the meristem. They showed that a fusion of the *Hd3a* promoter to the GUS marker gene was expressed in the phloem and xylem parenchyma tissue, but not at the shoot meristem (Tamaki *et al.*, 2007). By contrast, when Hd3a:GFP fusion protein was expressed from the *Hd3a* promoter it complemented the *hd3a* mutation and the protein was detected in the shoot meristem as well as the vascular tissue. Similarly, expression of Hd3a:GFP fusion protein from the *rolC* or *RPP16* promoters, which are active in the phloem, caused early flowering and the protein was found to accumulate in the shoot apical meristem. These results suggested that Hd3a protein could move from the phloem to the shoot meristem and the authors concluded that ‘Hd3a fulfills the requirements for a florigen’ (Tamaki *et al.*, 2007). In 2007, this groundbreaking discovery identified Hd3a protein as a mobile signalling molecule capable of inducing flowering, and has been supported by studies in *Arabidopsis*, pumpkin, Brassicas, and tomato (reviewed by Kobayashi and Weigel, 2007; Turck *et al.*, 2008). ‘That was an exciting year in the lab’ — recalls Hiroyuki Tsuji — ‘Ko encouraged his associates every day and enthusiastically discussed the results leading to the important conclusion of the mobile nature of Hd3a protein’.

FT proteins are related to lipid-binding proteins in animals, but in plants regulate transcription of target genes by interacting with transcription factors such as FD. Ko’s group strengthened this model by helping crystallize a complex of Hd3a and a rice bZIP transcription factor, OsFD1, which is related to FD of *Arabidopsis* (Taoka *et al.*, 2011). In this work, they demonstrated that *in vitro* Hd3a does not interact directly with OsFD1 unless a 14-3-3 protein is included in the reaction. This result suggested that the 14-3-3 protein bridges the interaction between Hd3a and OsFD1, and that in yeast this bridging function is probably mediated by yeast 14-3-3 proteins. The crystal structure of the Hd3a–14-3-3–OsFD1 complex was determined and found to be a heterohexamer containing two molecules each of Hd3a, 14-3-3, and OsFD1. Assays performed in protoplasts demonstrated that Hd3a interacts with the 14-3-3 protein to be translocated to the nucleus where it interacts with OsFD1. This complex can then bind to DNA and recognizes a motif in the promoter of the *OsMADS15* gene that encodes a MADS box transcription factor closely related to APETALA1, which confers floral meristem identity in *Arabidopsis* (Taoka *et al.*, 2011). The 14-3-3 protein was therefore defined as functioning as an intracellular receptor for Hd3a. Because Ko was enthusiastic to incorporate novel techniques in his lab, he combined novel cell-biology techniques, structural biology, biochemistry, and molecular genetics to reveal the protein complex containing Hd3a. These highly influential experiments provide a structural and mechanistic basis for the action of FT-like proteins and demonstrate how they act to regulate gene expression.

Thus, over the 8 years between the appearance of the Hayama *et al.* (2003) and Taoka *et al.* (2011) papers, the work of Ko and his collaborators dramatically changed our perception of photoperiodic flowering. They helped provide a genetic and mechanistic framework for understanding short-day responses in rice, but more radically identified FT-like proteins as mobile signals and provided a structural understanding of how they act to regulate gene expression. These major contributions provide a basis for future groups to follow Ko’s lead and provide an understanding of how the changes in gene expression induced by FT-like proteins change the developmental identity of organs.

Ko was a good example that brilliant scientists excel also at attracting great talents to their labs. Among the members who made important contributions to the progression of the flowering field in the Shimamoto laboratory, we have to mention Shuji Yokoi, Shojiro Tamaki, Takeshi Izawa, Ryo Ishikawa, Reina Komiya, Ryosuke Hayama, and Yasuyuki Takahashi.

## Other contributions

Other developmental and physiological responses are controlled by photoperiod in addition to flowering. These include tuberization of potato and bud dormancy of trees. Ko’s group collaborated with that of Salomé Prat to examine whether FT-like proteins control tuberization in potato (Navarro *et al.*, 2011). Normally, potato tubers are formed in response to short days but they showed that transgenic potato carrying the *RolC:Hd3A* transgene formed tubers under non-inductive long days. This observation suggested that FT-like proteins do control this photoperiodic response from the leaves. Consistent with this idea, they identified the potato *FT*-like gene *StSP6a* as being transcriptionally induced in response to short days in leaves and stolons and to cause constitutive tuberization when overexpressed. Grafting experiments indicated that expression of the *FT*-like genes *Hd3a* or *StSP6a* in the leaves caused local induction of *StSP6a* in the stolons where tubers develop. Thus, here amplification of the mobile signal after arrival in the stolon is an important aspect of the model (Navarro *et al.*, 2011). This paper showed that *FT*-like genes are important in other photoperiodic responses apart from flowering and that they can influence the development of organs other than the shoot apical meristem. These results, together with those of other groups studying bud dormancy in poplar, led to the idea that FT-like proteins are probably implicated in all photoperiodic responses in plants.

The breadth of Ko’s scientific achievements is difficult to encompass. Sometimes through collaborations with other laboratories, sometimes through the work of his own group, he made contributions to countless other aspects of plant biology, including other developmental events, e.g. shoot branching; several aspects of gene silencing, such as the role of small interfering RNAs in chromatin modifications and the influence of the RNA silencing machinery on virus propagation; RNA splicing; retrotransposons; brassinosteroid homeostasis; and photosystem function. He also developed tools for the study of rice, such as methods for transformation and plant regeneration from protoplasts, a transposon tagging system and vectors for RNA interference. In his last days, another manuscript was edited and completed from his sickbed that will be published in the near future.

During his career Ko received many honors, including the Japanese Society of Breeding Award (1993), the Kihara Memorial Foundation Prize (2000), the Prize for Science and Technology from Japan’s Ministry of Education, Culture, Sports, Science, and Technology (2011), and the prestigious Purple Ribbon Medal of Honor from the Japanese Government (2012).

Ko loved playing the violin, skiing, and of course, karaoke. Last autumn his lab held a party to celebrate the rice harvest from the university’s experimental plots. As usual, Ko played beautiful violin music for all the lab members at that party. In winter, Ko loved to ski and fondly remembered the days in Switzerland that provided a great opportunity for both research and skiing. He often invited the lab students to his private cottage near a ski resort to enjoy skiing. Spring was the season for students and postdocs to graduate or to move to other labs in Japan, and Ko’s karaoke was always the highlight of the farewell party for these lab members. All these experiences were quite impressive; Ko was a great mentor to young students and lab researchers.

He was also very supportive of scientists working in other laboratories. ‘My professional relationship with Ko’ –— recalls Paula Suárez-López –— ‘had a very special touch of kindness, warmth and generosity. I met him for the first time at an international conference, when I was a postdoc. He came to my poster and we discussed results that, although I was convinced they could have interesting implications, at that time had been received with certain scepticism. To my pleasant surprise, Ko showed much interest and encouraged me to follow that path. From that meeting on, every time we met at a conference, Ko greeted me with a cheerful smile and we discussed openly the latest results from his laboratory, as well as my results. Speaking with him was always one of the most enjoyable and encouraging moments in scientific conferences’.

Ko enjoyed showing his guests around the ancient city of Nara, with its beautiful historic wooden buildings. ‘I had the pleasure to meet Ko at many international meetings, to host him in Cologne and to be hosted twice by him in Nara’ remembers George Coupland. ‘He was always an entertaining, motivated, and friendly companion. In Nara, he was a generous host, and I remember him taking a small group of us one November to see the autumn colours of maple trees spectacularly illuminated after dark’.

Ko’s death has deeply saddened his colleagues, friends, former and present students, and also the scientific community. We have lost him far too early, and while he was still very actively contributing to our field. He will be remembered as an innovator in plant molecular genetics. We are grateful for the opportunity to experience his enthusiasm for plant science and will remember him with loving memories.

*Shimamoto-san, Arigato*
